# Using the Social Robot NAO for Emotional Support to Children at a Pediatric Emergency Department: Randomized Clinical Trial

**DOI:** 10.2196/29656

**Published:** 2022-01-13

**Authors:** Silvia Rossi, Silvano Junior Santini, Daniela Di Genova, Gianpaolo Maggi, Alberto Verrotti, Giovanni Farello, Roberta Romualdi, Anna Alisi, Alberto Eugenio Tozzi, Clara Balsano

**Affiliations:** 1 Department of Electrical Engineering and Information Technology University Federico II-Naples Naples Italy; 2 Department of Life, Health & Environmental Sciences School of Emergency and Urgency Medicine University of L'Aquila L'Aquila Italy; 3 Department of Psychology University of Campania “Luigi Vanvitelli” Caserta Italy; 4 Department of Pediatrics University of Perugia Perugia Italy; 5 Pediatric Unit Department of Life, Health & Environmental Sciences University of L'Aquila L'Aquila Italy; 6 Research Unit of Molecular Genetics of Complex Phenotypes Bambino Gesù Children’s Hospital Istituto di Ricovero e Cura a Carattere Scientifico Rome Italy; 7 Multifactorial and Complex Diseases Research Area Bambino Gesù Children's Hospital Istituto di Ricovero e Cura a Carattere Scientifico Rome Italy

**Keywords:** children, emotional health, emergency department, social robots, anxiety, stress

## Abstract

**Background:**

Social robots (SRs) have been used for improving anxiety in children in stressful clinical situations, such as during painful procedures. However, no studies have yet been performed to assess their effect in children while waiting for emergency room consultations.

**Objective:**

This study aims to assess the impact of SRs on managing stress in children waiting for an emergency room procedure through the assessment of salivary cortisol levels.

**Methods:**

This was an open randomized clinical trial in children attending a pediatric emergency department. Children accessing the emergency room were randomized to 1 of 3 groups: (1) playing with a NAO SR, (2) playing with a study nurse, or (3) waiting with parents. The salivary cortisol levels of all children were measured through a swab. Salivary cortisol levels before and after the intervention were compared in the 3 groups. We calculated the effect size of our interventions through the Cohen *d*-based effect size correlation (*r*).

**Results:**

A total of 109 children aged 3-10 years were enrolled in the study, and 94 (86.2%) had complete data for the analyses. Salivary cortisol levels significantly decreased more in the group exposed to robot interaction than in the other two groups (*r*=0.75). Cortisol levels decreased more in girls (*r*=0.92) than in boys (*r*=0.57).

**Conclusions:**

SRs are efficacious in decreasing stress in children accessing the emergency room and may be considered a tool for improving emotional perceptions of children and their families in such a critical setting.

**Trial Registration:**

ClinicalTrials.gov NCT04627909; https://clinicaltrials.gov/ct2/show/study/NCT04627909

## Introduction

Social robots (SRs) may offer a multifactorial sensory experience to children and distract them from stressful situations, as it frequently happens during health care encounters [1,2]. SRs are designed to interact and communicate with human beings by play, gestures, poses, gaze, and colors and have been successfully used with pediatric patients in different settings [3,4], although not all the available devices on the market have anthropomorphic, physical, and behavioral qualities to establish a virtuous collaboration with children [5-8].

Addressing the emotional needs of children who present to the pediatric emergency department (ED) is a complex task that requires the management of anxiety and pain during medical procedures [9]. In such situations, emotional stress may affect the outcome of emergency interventions due to the lack of cooperation of young patients with health care providers, may delay the diagnosis, and may prolong medical procedures [10,11]. For this reason, interventions for reducing stress in children attending the ED are highly desirable [12-14].

Although several studies have explored the impact of SRs on negative emotions in children [3,15,16], their efficacy in reducing stress in hospitalized children is still uncertain. Moreover, to the best of our knowledge, the impact of SRs on stress in children accessing the ED has not been investigated yet.

We therefore conducted a randomized clinical trial with the aim of comparing SR interaction with playing with a nurse, or no intervention, in children accessing the emergency room prior to entering the medical office. Our hypothesis was that SR interaction is superior to other interventions in reducing stress. As the biological response in stressful situations includes the activation of the hypothalamic-pituitary-adrenal axis, a glucocorticoid response and subsequent cortisol release [17], we measured as an outcome salivary cortisol levels in children.

## Methods

### Study Design and Participants

This was an open, 3-arm, parallel, randomized clinical trial conducted on children who attended the pediatric ED of the San Salvatore Hospital of L’Aquila from September 1, 2019, to February 29, 2020. Children eligible for the trial were those 3-10 years old, who were assigned a white (not critical), green (not very critical), or yellow (moderately critical) code with no neurological condition at triage, as described by Piccotti et al [18], and who could safely wait for nonurgent care in the waiting room. Exclusion criteria were age range <3 and >10 years; parents not fluent in Italian; yellow code for headache due to recent trauma with visual disturbances, headache accompanied by neck rigidity, vomiting, or indifference to the environment; presence of dyspnea; significant trauma to the head with an altered state of consciousness; and red code (very critical).

Parents and children who attended the pediatric ED of the San Salvatore Hospital of L’Aquila in the study period were invited to participate in the study, and informed consent (parent) and assent (child) were obtained. Enrolment was restricted between 8:30 AM and 10:30 AM to avoid the differences in cortisol levels due to their circadian fluctuation. The children were then randomized to 1 of 3 groups: (1) interaction with an SR, (2) playing with a study nurse, or (3) a control group assigned to routine waiting with parents. For all the 3 groups, there were no restrictions regarding whether the parents could stay with their children; however, only children in the control group could interact with them. All children in the study received standard care.

**Figure 1:**
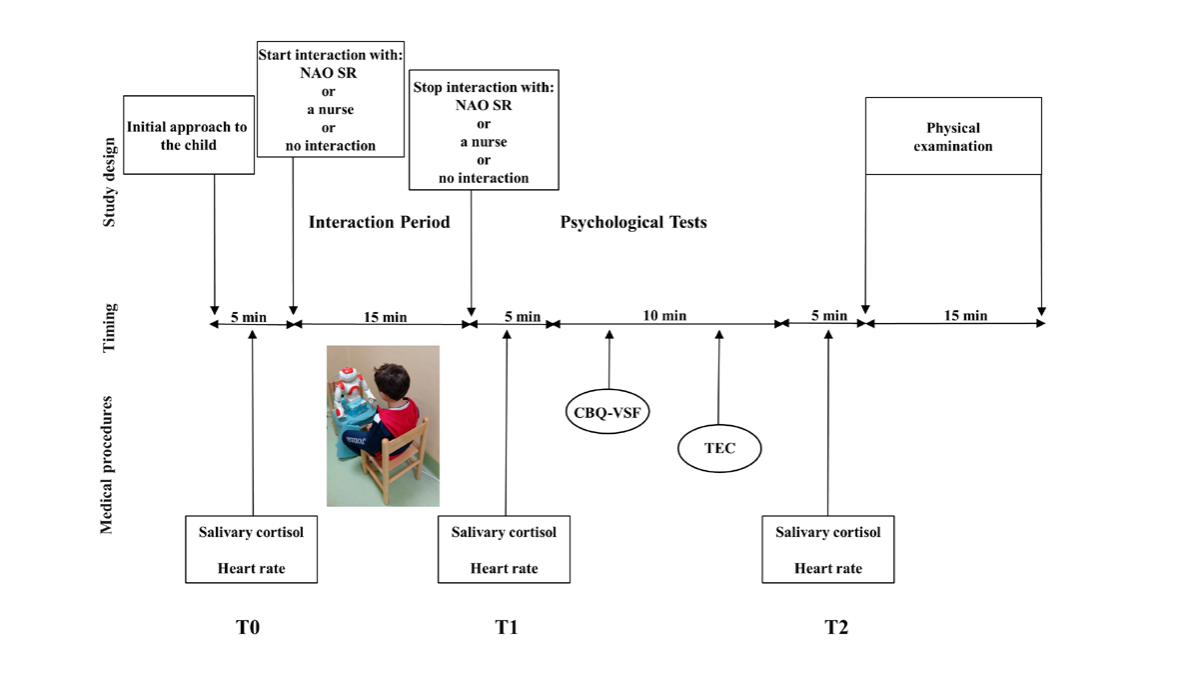
Cohort assembly: study procedure and timing of outcome assessment. CBQ-VSF: Children’s Behavior Questionnaire-Very Short Form; T0-T1-T2: timing of cortisol level measurement; TEC: Test of Emotion Comprehension.

Once randomized, the children’s temperature, heart rate, and a salivary sample were taken (T0). The children were then exposed to 1 of the 3 interventions for 15 minutes. Immediately after the intervention, the children’s heart rate was measured again and an additional salivary sample was taken (T1). After that, the children underwent a battery of psychological tests (see the Interventions section), which took nearly 10 minutes to complete, and a third heart rate measure and a salivary sample were taken thereafter (T2). Finally, the children entered the medical office in the ED. The entire process is illustrated in Figure 1.

The primary outcome measure of the study was the difference in cortisol level of patients before (T0) and after the intervention (T2).

### Interventions

The NAO robot is a small SR, developed by Softbank Robotics, programmed to interact with children (Figure 1) [10,11]. The robot uses cognitive-behavioral distraction strategies appropriate to age (songs, stories, jokes, games, riddles) with children [19-22]. The robot started asking the child’s name and age. The robot then autonomously selected the appropriate interaction according to age. Questions posed by the robot to tailor interactions included school attendance, ability to count, favorite subjects, and cartoons. Based on this information, the robot asked the child to count together and to talk about school and favorite subjects and cartoons. A further interpretation of the answers provided by children allowed the robot to make comments and to play the theme songs of the cartoon. The robot also played songs and jokes appropriate for the children’s age, inviting them to guess the title or the solution. An additional interaction was about nursery rhymes and tongue twisters. Finally, the robot played guessing games about animal noises and other riddles.

The NAO robot was equipped with natural language processing technologies to understand the child's speech. However, in the case of background noise or incorrect pronunciation, a doctor was present to enter manually answers into the NAO software by the use of a hidden personal computer.

In the second intervention group, children played with nurses by coloring children’s books or using toys available in the waiting room.

Children in the control group stayed with their parents and were free to play while waiting.

### Salivary Analysis and Heart Rate Measurement

Nurses who were not directly involved in this study measured the children’s heart rate and temperature.

Salivary samples were collected after rinsing the mouth with water to prevent food contamination. A swab from a Salivette device (cat. 51.1534, Sarstedt, Nümbrecht, Germany) was placed under the tongue of the participant, and saliva was absorbed for 60 seconds. The saliva-saturated Salivette swab then was placed in a polypropylene tube and centrifuged at 1000 ×*g* for 15 minutes at 4°C for saliva extraction. The saliva sample was then frozen at −20°C. To start the analysis of the salivary cortisol levels, the saliva was centrifuged at 2500 ×*g* for 20 minutes after thawing, and the clear supernatant was used in the analysis.

The salivary cortisol levels were assessed using a commercially available DetectX Cortisol Enzyme Immunoassay Kit (cat. K003-H1W, Arbor Assays, MI, USA) and a Victor3 microplate reader (PerkinElmer, Waltham, MA, USA) according to the manufacturers’ instructions.

### Psychological Tests

To verify that salivary cortisol levels may have been not confounded by temperament or emotional management, we performed a series of psychological tests at T1. Although this evaluation was performed after the intervention to avoid interferences with the study and the ED workflow, it is important to ensure that the tests performed at this time are not influenced by external stimuli, including those of the intervention. For this reason, these tests were considered useful for comparing participants at baseline. The evaluation consisted of the Children’s Behavior Questionnaire-Very Short Form (CBQ-VSF), which evaluates children’s temperament, and the Test of Emotion Comprehension (TEC), which were administered and completed in the presence of assistant psychologists.

The CBQ-VSF [23,24] is a 36-item questionnaire that is completed by the child caregiver to assess the temperament of children aged 3-8 years. It is designed to measure 3 broad dimensions: surgency/extraversion, negative affectivity, and effortful control. The surgency/extraversion scale is characterized by high activity levels, high-intensity pleasure seeking, and low shyness and impulsivity. The second dimension, negative affectivity, is defined by feelings of sadness, discomfort, frustration, and fear. The effortful control scale encompasses inhibitory control, attentional focus, low-intensity pleasure, and perceptual sensitivity [25]. Caregivers were asked to rate how well the items describe the child’s reaction in a variety of situations. The responses were given on a 7-point scale ranging from 1 (extremely untrue of my child) to 7 (extremely true of my child).

The TEC [26] evaluates the understanding and managing of emotions in children aged 3-11 years. It consists of 9 components, namely the ability of recognition of emotions based on facial expressions (labeling), the comprehension of external emotional causes, the impact of desire on emotions, emotions based on beliefs, the influence of memories on emotions, the possibility of emotional regulation, the possibility of hiding an emotional state, having mixed emotions, and the contribution of morality to emotional experiences [27].

### Statistical Analysis and Sample Size

Randomization of the intervention was made through computer-generated randomization codes (Random Allocation Software version 1.0, Isfahan University of Medical Sciences) by an independent researcher, and an envelope containing sequential numbers was given to the parents.

Data for all variables were checked for normal distribution through the Shapiro-Wilk test. As the test indicated that distributions were not normally distributed, we used medians and IQRs for continuous variables. To evaluate the differences in cortisol levels and heart rate at the 3 different time points within each group, we used the Friedman nonparametric test. Post hoc analyses with the Wilcoxon signed-rank test were carried out to determine where the observed differences were. To evaluate the strength of the relationship between the variables of our interest, we calculated the effect size of our interventions through the Cohen *d*-based effect size correlation (r).

Linear regression analyses were carried out to evaluate the relationship between salivary cortisol levels and heart rate registered at each time within each age group.

Sample size was calculated based on variations of cortisol levels. It was estimated that to detect a reduction in cortisol levels of at least 0.6 ng/mL from T0 to T2, assuming an SD of 0.17 for cortisol levels, with a 95% statistical power (CI, Z=1.96; proportion of the population, π=0.80; margin of error E=5%, prevalence control group, *P*_0_=.85, prevalence health care personnel group, *P*_1_=.75, NAO robot group, *P*_2_=.80), we evaluated 19 children in each group.

All statistical analyses were performed using Graphpad Prism version 9.0.

The trial was approved by the local ethics committee of the San Salvatore Hospital of L’Aquila (IRB protocol no. 2666 06-25-2019) and registered on ClinicalTrials.gov (identification no. NCT04627909).

## Results

### Demographic, Psychological, and Other Baseline Characteristics

We invited 145 consecutive families whose children were eligible to participate in the study, and we enrolled a total of 109 patients. Reasons for refusing participation were (1) parents did not want to let their children interact with NAO (n=15, 42%), parents thought that their children were already overexposed to electronic devices (n=8, 22%), parents were particularly concerned about their child's illness (n=7, 19%); and consent by both parents could not be obtained (n=6, 17%).

**Figure 2:**
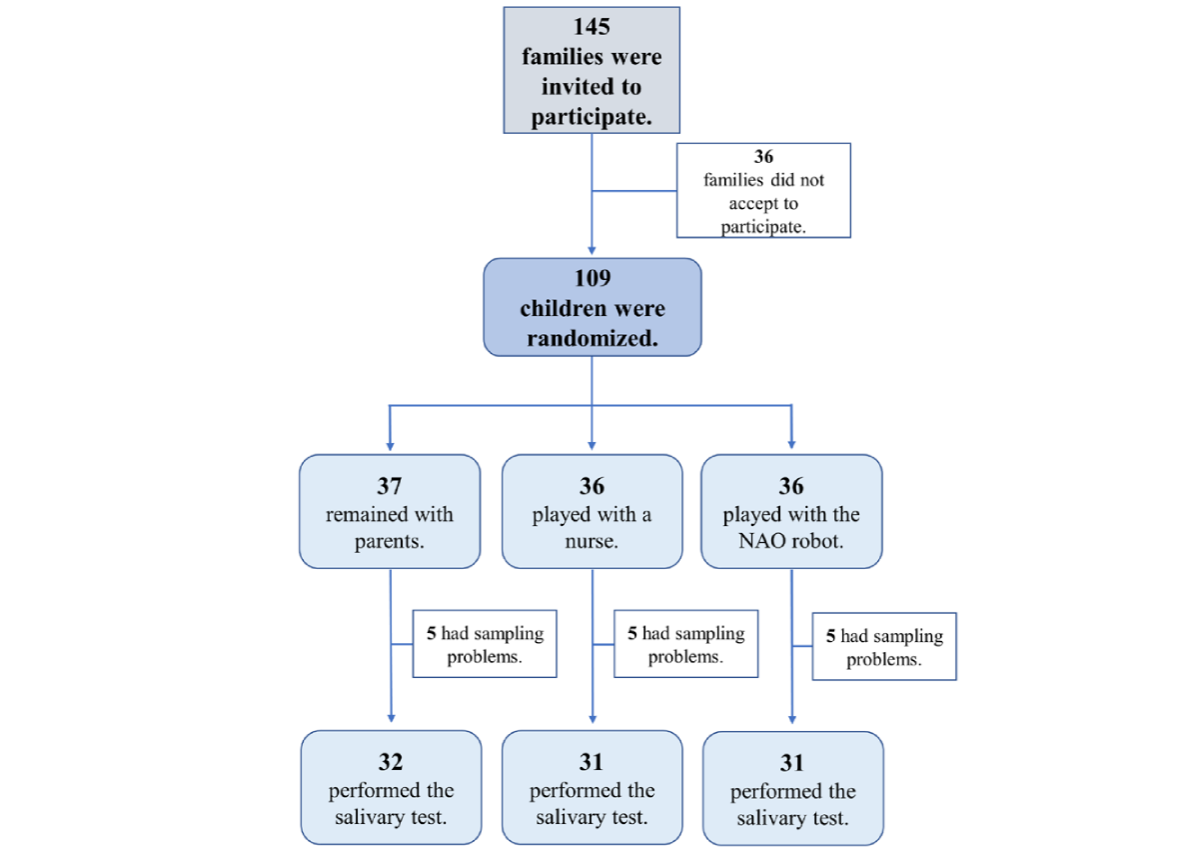
Enrollment and randomization of children.

Of the 109 children, 94 (86.2%) were included in the final analysis as 5 salivary samples in each of the 3 groups could not be analyzed because of sampling problems. Among them, 32 (34%) were in the NAO robot group, 31 (33%) were in the group playing with a study nurse, and 31 (33%) remained with their parents (Figure 2).

A description of demographic, psychological, and other baseline characteristics of children by intervention group are reported in Tables 1 and 2 and in Multimedia Appendices 1 and 2. The baseline characteristics of children in the 3 groups were balanced and did not show significant differences.

Of all 94 participants, 48 (51%) were boys. There were no significant differences in the salivary cortisol levels, heart rate, and temperature among the groups at T0 (Table 1). The average total score of the TEC for children interacting with the NAO robot was 6.8 (2.0), while it was 6.5 (1.2) in the group of children playing with nurses and 6.3 (2.0) in the control group (Table 2). The temperaments of the 3 groups evaluated using the CBQ-VSF displayed similar profiles for extraversion/surgency, negative affectivity, and effortful control; the distribution of the scores is reported in Table 2. These results were obtained in 71 of 94 (76%) children only, because 23 children (24%) did not complete the tests due to ED time constraints.

**Figure 3:**
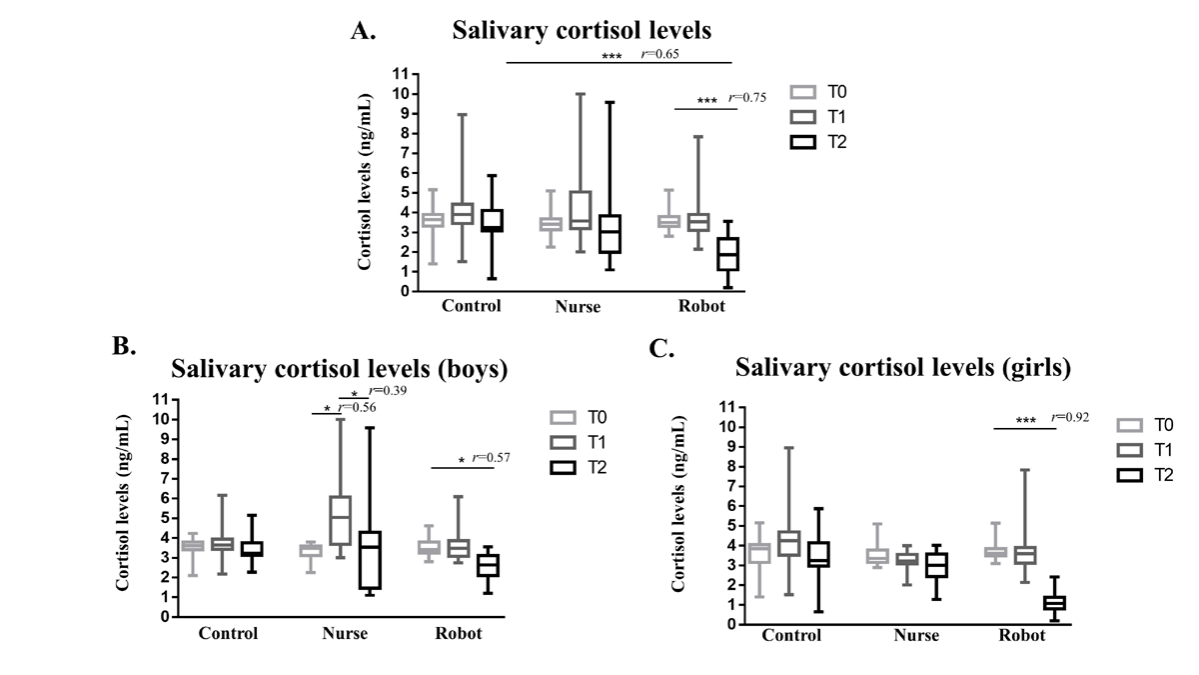
Salivary cortisol levels (A) in the whole sample, (B) in boys, and (C) in girls. Data are expressed as the median and IQR (*P<.05, **P<.01, ***P<.001). *r* represents the Cohen *d*-based effect size correlation.

The lowest cortisol levels at T2 were measured in children with NAO robot interaction (Figure 3A). In this group, cortisol levels at T2 significantly differed from those at T0 (3.50 vs 1.87 ng/mL, *P*<.001; Figure 3A). In addition, cortisol levels slightly decreased at T2 in children playing with nurses, and they were similar at T0 and T1 in all groups.

When exploring differences in salivary cortisol levels by sex, we found a more pronounced decrease at T2 in girls interacting with the SR, than in boys (Figure 3A-C). The trend was different in boys playing with a nurse, who showed an increase in cortisol levels at T1, which returned to levels similar to baseline at T2, while no significant variations were observed in the control group (Figure 3B).

**Figure 4:**
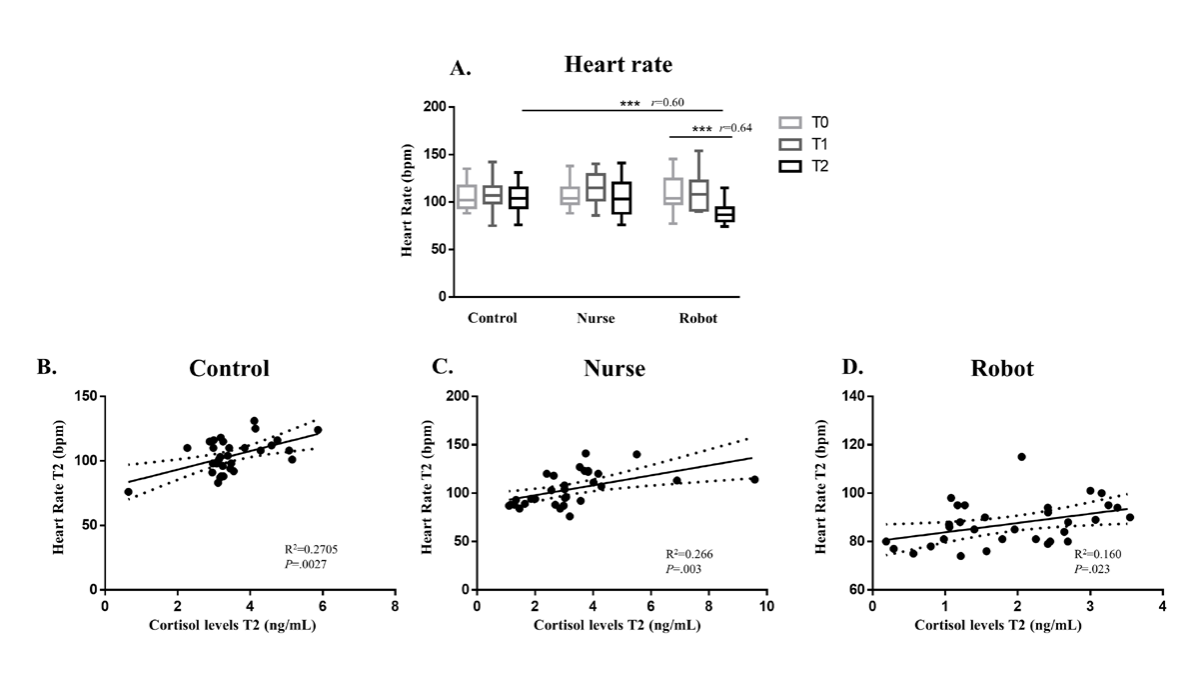
Heart rate (A) in the whole sample. Data are expressed as the median and IQR (***P<.001; r represents the Cohen *d*-based effect size correlation). Direct linear regression between heart rate and salivary cortisol levels at T2 in the control group (B), study nurse group (C), and NAO robot group (D).

When looking at the trends in the heart rate overall, we found the same differences observed for salivary cortisol levels (Figure 4A). Moreover, regression analysis showed a significant linear correlation between salivary cortisol levels and heart rate in all intervention groups (Figure 4B-D).

**Table 1:** Demographics by group.

Demographics		All groups (N=94)	Control group (n=31)	Study nurse group (n=31)	NAO robot group (n=32)
Age, median (IQR)		7 (5-8)	7 (6-7.5)	7 (5.5-8)	6 (5-7)
**Sex, n (%)**					
	Boys	48 (51.1)	16 (51.6)	15 (48.4)	17 (53.1)
	Girls	46 (48.9)	15 (48.4)	16 (51.6)	15 (46.9)
**Triage code, n (%)**					
	White	0	0	0	0
	Green	90 (95.7)	30 (96.8)	30 (96.8)	30 (93.7)
	Yellow	4 (4.3)	1 (3.2)	1 (3.2)	2 (6.3)
**Discharge, n (%)**					
	Home discharge	91 (96.8)	30 (96.8)	30 (96.8)	31 (96.9)
	Hospitalization	3 (3.2)	1 (3.2)	1 (3.2)	1 (3.1)
T0 heart rate (bpm), median (IQR)		103 (97.25-117)	102 (95-116)	104 (98-114.5)	105 (99-123.5)
T0 cortisol levels (ng/mL), median (IQR)		3.54 (3.26-3.80)	3.65 (3.35-3.89)	3.41 (3.13-3.67)	3.51 (3.33-3.80)
Temperature (°C), median (IQR)		36.4 (36-36.9)	36.5 (36.2-36.9)	36.2 (36.0-36.5)	36.5 (36-36.9)

**Table 2:** Psychological variables by group.

Psychological variables			Total sample (N=71)		Control group (n=19)	Study nurse group (n=23)		NAO robot group (n=29)
**TEC^a^, mean (SD)**								
	Total score	6.6 (1.8)		6.3 (2.0)		6.5 (1.2)	6.8 (2.0)	
	Component^b^ I	1.0 (0.2)		1.0 (0.0)		1.0 (0.2)	1.0 (0.2)	
	Component II	0.9 (0.3)		0.8 (0.4)		0.9 (0.3)	0.9 (0.3)	
	Component III	0.9 (0.3)		0.9 (0.3)		0.8 (0.4)	0.9 (0.3)	
	Component IV	0.7 (0.5)		0.6 (0.5)		0.7 (0.5)	0.8 (0.4)	
	Component V	0.8 (0.4)		0.6 (0.5)		0.9 (0.3)	0.8 (0.4)	
	Component VI	0.5 (0.5)		0.4 (0.5)		0.4 (0.5)	0.5 (0.5)	
	Component VII	0.7 (0.4)		0.8 (0.4)		0.7 (0.5)	0.7 (0.5)	
	Component VIII	0.4 (0.5)		0.4 (0.5)		0.3 (0.5)	0.5 (0.5)	
	Component IX	0.7 (0.4)		0.7 (0.5)		0.8 )0.4)	0.8 )0.4)	
**CBQ-VSF^c^, mean (SD)**								
	Extraversion/surgency	52.8 (8.4)		57.3 (6.3)		49.7 (9.8)	53.1 (7.5)	
	Negative affectivity	51.0 (8.0)		55.6 (7.0)		51.6 (6.8)	49.1 (8.6)	
	Effortful control	66.2 (8.1)		61.2 (8.1)		65.4 (8.1)	68.3 (7.4)	

^a^TEC: Test of Emotion Comprehension.

^b^Component I: recognition of emotions; component II: comprehension of external emotional causes; component III: impact of desire on emotions; component IV: emotions based on beliefs; component V: memory influence on emotions; component VI: possibility of emotional regulation; component VII: possibility of hiding an emotional state; component VIII: mixed emotions; component IX: contribution of morality to emotional experiences.

^c^CBQ-VSF: Children’s Behavior Questionnaire-Very Short Form.

## Discussion

### Principal Findings

Our study shows that playing with an SR while waiting for a medical procedure in the emergency room may decrease the stress level in children, as demonstrated by a decrease in salivary cortisol levels. Our study shows that this effect is visible not immediately after the intervention but nearly 20 minutes after robot interaction, according to the physiological cortisol response to stressor stimuli changes that occur 15-30 minutes after stressor onset [28].

Moreover, the decrease in cortisol levels is more evident in girls than in boys.

Sex differences in the attitude to interacting with SRs have been already reported, and a previous study conducted in Japan suggested that females have more negative attitudes than males toward robot interaction [29,30]. Although our findings are in contrast with these observations, this is not surprising as cultural background, setting, and perceived stereotypes may affect the attitude of children in using technology [29,31-34].

A recent systematic review examined the effect of SRs on anxiety and distress in children, and 4 of the 10 (40%) included studies used the NAO robot, the same device as in our study [3]. SRs reduced distress and anxiety in all reviewed studies, although the quality of evidence was low mainly due to the small sample size. Our results are in line with the published literature; however, we are not aware of any study on the effect of SRs in children conducted in the emergency room setting. Moreover, differently from the available studies in the literature, we used salivary cortisol levels as an outcome, as they may be measured through an easy and noninvasive method [35-37] and are associated with stress from nonpharmacological interventions [36,38-40].

We administered psychological tests to assess the temperament of children that might affect the control of anxiety in distressing situations. Higher scores on surgency/extraversion temperament have an impact on the cortisol production independently from the assigned intervention arm, demonstrating that children with more extroverted personalities have less emotional distress at baseline. These findings further support the idea that emotion regulation abilities contribute to reduced fear and anxiety in distressing situations [37,38,41,42].

More than 15% (6/36) of parents of children, eligible for our study, refused to participate in the study because they were concerned about exposure of their children to electronic devices. Although setting and cultural background may play a role, this observation should be considered if strategies including SRs for reducing stress and anxiety will be put in place in routine clinical practice.

### Strengths and Limitations

This study had several strengths. Its design prevented common confounding effects, as shown by the baseline characteristics of participants that were similar across intervention groups. Moreover, we used salivary cortisol levels to assess the stress level in children, which represents an objective measure of this outcome. To validate our results, we also studied the correlation between salivary cortisol levels and heart rate.

A limitation was that our study was conducted in a single clinical center and we did not administer any psychological test to assess stress/anxiety to avoid slowing down medical procedures in the emergency room. However, psychological questionnaires are less reliable than salivary cortisol levels in detecting slight differences in children’s stress levels.

Although SRs represent a promising tool in managing children’s distress in the emergency room, the cultural background and setting may strongly affect the acceptance of these devices. However, we frequently noticed positive comments from parents, such as “My son didn’t want to leave the NAO robot,” “My son wanted to take the robot with him,” “My son told me that the NAO robot was amazing,” and “My son asked, for the first time, to come back again to the ED.” Additional studies may better address determinants of acceptability in different settings.

### Conclusion

In conclusion, our study demonstrates that SRs are effective in decreasing the stress of children in health care emergency contexts. These devices may be integrated in the pediatric ED workflow, with benefits for patients and families and potentially to speed up clinical procedures. To this aim, future and larger studies in different settings should be promoted. Moreover, in circumstances where social contacts should be prevented, such as during the COVID-19 pandemic, SRs may play an important role in improving the emotional experiences of children and their families, and disease outcomes.

## Appendix

Multimedia Appendix 1 Psychological features of children. (A) TEC and (B-D) tests. Data are shown as the mean and SD. Statistically significant differences in the TEC and CBQ scores between groups were not detected. CBQ: Children’s Behavior Questionnaire; TEC: Test of Emotion Comprehension.

Multimedia Appendix 2 Vital signs of children. Heart rate (A) and temperature (B) at T0. Data are expressed as the median and IQR. No statistically significant differences in the TEC and CBQ between groups were detected. CBQ: Children’s Behavior Questionnaire; TEC: Test of Emotion Comprehension.

Multimedia Appendix 3 CONSORT-eHEALTH checklist (V 1.6.1).

## Abbreviations

CBQ-VSF: Children’s Behavior Questionnaire-Very Short Form
ED: emergency department
SR: social robot
TEC: Test of Emotion Comprehension
