# Supplementary figures and images for: Using the Social Robot NAO for Emotional Support to Children at a Pediatric Emergency Department: Randomized Clinical Trial
Source: J Med Internet Res. 2022 Jan 13;24(1):e29656. doi: 10.2196/29656 (PMC8796042; doi:10.2196/29656)

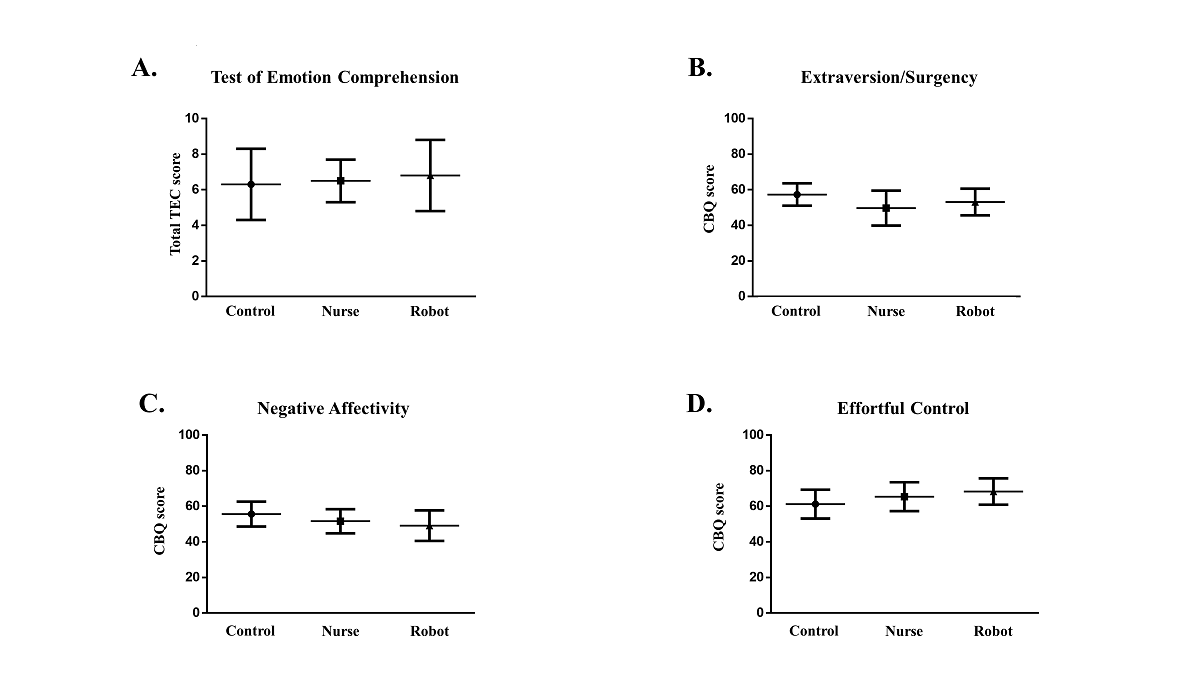

Supplement: Multimedia Appendix 1 [file jmir_v24i1e29656_app1.png]

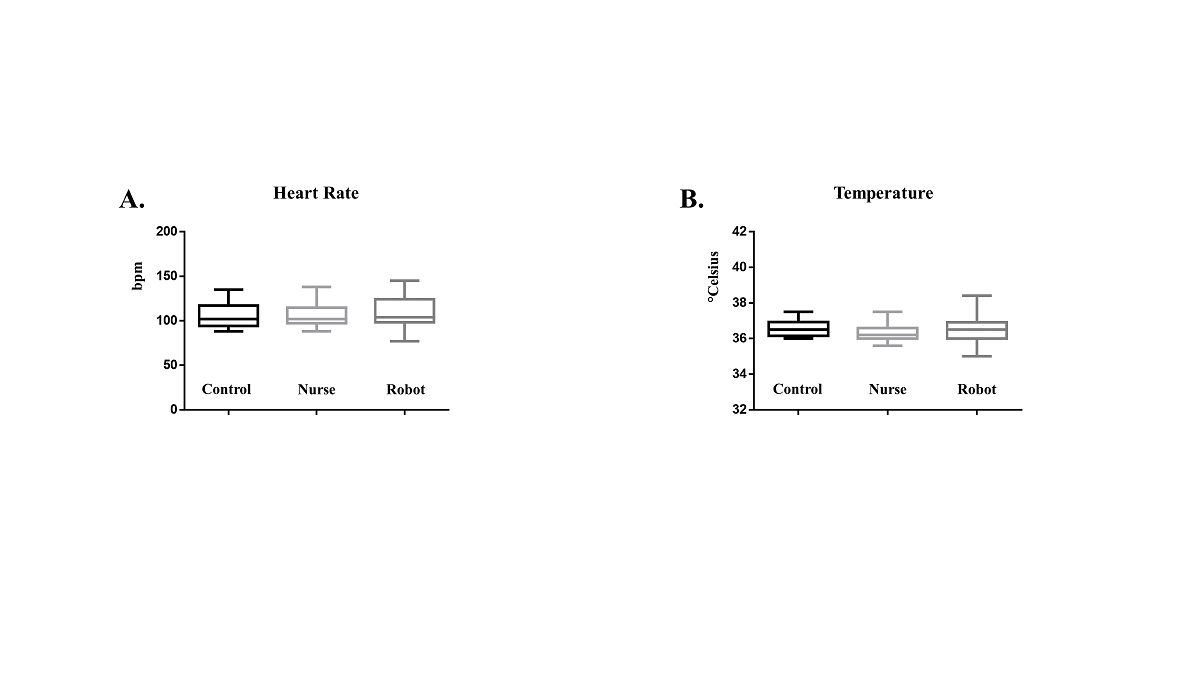

Supplement: Multimedia Appendix 2 [file jmir_v24i1e29656_app2.png]
